# A pumpless monolayer microfluidic device based on mesenchymal stem cell-conditioned medium promotes neonatal mouse in vitro spermatogenesis

**DOI:** 10.1186/s13287-023-03356-x

**Published:** 2023-05-11

**Authors:** Selin Önen, Ali Can Atik, Merve Gizer, Sevil Köse, Önder Yaman, Haluk Külah, Petek Korkusuz

**Affiliations:** 1 Department of Stem Cell Sciences, Hacettepe University, Ankara, Turkey; 2 Department of Medical Biology, Atilim University, Ankara, Turkey; 3 Department of Electrical and Electronics Engineering, Middle East Technical University, Ankara, Turkey; 4 METU MEMS Center, Ankara, Turkey; 5 Department of Plastic, Reconstructive, and Aesthetic Surgery, Akdeniz University, Antalya, Turkey; 6 Department of Urology, Ankara University, Ankara, Turkey; 7 Department of Histology and Embryology, Faculty of Medicine, Hacettepe University, Sihhiye, Ankara, 06100 Turkey

**Keywords:** In vitro spermatogenesis, Microfluidics, Mesenchymal stem cells, Male infertility, Prepubertal boys

## Abstract

**Background:**

Childhood cancer treatment-induced gonadotoxicity causes permanent infertility/sub-infertility in nearly half of males. The current clinical and experimental approaches are limited to cryopreservation of prepubertal testicular strips and in vitro spermatogenesis which are inadequate to achieve the expanded spermatogonial stem/progenitor cells and spermatogenesis in vitro. Recently, we reported the supportive effect of bone marrow-derived mesenchymal cell co-culture which is inadequate after 14 days of culture in static conditions in prepubertal mouse testis due to lack of microvascular flow and diffusion. Therefore, we generated a novel, pumpless, single polydimethylsiloxane-layered testis-on-chip platform providing a continuous and stabilized microfluidic flow and real-time cellular paracrine contribution of allogeneic bone marrow-derived mesenchymal stem cells.

**Methods:**

We aimed to evaluate the efficacy of this new setup in terms of self-renewal of stem/progenitor cells, spermatogenesis and structural and functional maturation of seminiferous tubules in vitro by measuring the number of undifferentiated and differentiating spermatogonia, spermatocytes, spermatids and tubular growth by histochemical, immunohistochemical, flow cytometric and chromatographic techniques.

**Results:**

Bone marrow-derived mesenchymal stem cell-based testis-on-chip platform supported the maintenance of SALL4(+) and PLZF(+) spermatogonial stem/progenitor cells, for 42 days. The new setup improved in vitro spermatogenesis in terms of c-Kit(+) differentiating spermatogonia, VASA(+) total germ cells, the meiotic cells including spermatocytes and spermatids and testicular maturation by increasing testosterone concentration and improved tubular growth for 42 days in comparison with hanging drop and non-mesenchymal stem cell control.

**Conclusions:**

Future fertility preservation for male pediatric cancer survivors depends on the protection/expansion of spermatogonial stem/progenitor cell pool and induction of in vitro spermatogenesis. Our findings demonstrate that a novel bone marrow-derived mesenchymal stem cell-based microfluidic testis-on-chip device supporting the maintenance of stem cells and spermatogenesis in prepubertal mice in vitro. This new, cell therapy-based microfluidic platform may contribute to a safe, precision-based cell and tissue banking protocols for prepubertal fertility restoration in future.

**Supplementary Information:**

The online version contains supplementary material available at 10.1186/s13287-023-03356-x.

## Background

The American Cancer Society estimated a 85% survival rate for around ten thousand prepubertal cancer patients being diagnosed in 2022 [1], of whom half will suffer from permanent infertility [2–5]. Spermatogonial stem/progenitor cells (SSPC) constitute the only source for fertility preservation since spermatogenesis is not initiated until puberty. In vitro spermatogenesis (IVS) from SSPCs is a challenging process that takes place in a complex 3D niche consisting of vascular flow sourcing mediators, accessory cells and stromal elements regulating paracrine and physical signals. Previously, we demonstrated the potential of a monolayer cell culture setup in prepubertal C57BL/6 mice SSPCs to initiate spermatogenesis [6]. However, human SSPCs display inadequate colonization in vitro when other niche components are absent [7].

Mouse [8–10], rat [11], goat [12] and human [13, 14] testicular organ cultures comprising niche components supported further differentiation up to spermatid state and improved testicular maturation in terms of tubular and luminal diameter and enlargement of the spermatogonial pool. Those experimental settings use a variety of supplements consisting of 10% KSR, AlbuMAX (10–40 mg/ml), hormones, antioxidants and lysophospholipids on 3D supporting air–liquid interphase (ALI) [9, 10, 15], hanging drop (HD) [12], soft agar [16] and alginate [8] matrixes. However, these systems have serious limitations in terms of inadequate accessory cell performance and also lack of physiologic microcirculation that could mimic the in vivo niche conditions.

Recently testis-on-chip microfluidics including pumpless two polydimethylsiloxane (PDMS) layered [17] and pumped with single [18] and double PDMS-layered [19] setups provided effective testosterone levels for improved spermatogenesis. Those systems require high performance of all niche components in addition to microvascular flow. Mesenchymal stem cells and their exosomes promote the restoration of spermatogenesis in non-obstructive azoospermia [20], abdominal cryptorchidism [21], busulfan [22, 23], cisplatin [24] or lead nitrate [25]-induced infertility and ischemia–reperfusion [26, 27] and torsion–detorsion injury [28, 29] in rodents in vivo. In a previous study from our laboratory, we demonstrated that syngeneic bone marrow-derived mesenchymal stem cells (BMSC) enlarged the SSPC pool and induced IVS up to the round spermatid stage when added to the static ALI co-culture platform [30]. The efficacy of the BMSC containing co-culture system, however, was limited due to the lack of microvascular flow and inadequate diffusion of nutrients in the medium. Therefore, ex vivo long-term spermatogenesis requires a dynamic platform simulating the microvascular 3D physiology of the testicular niche. We designed, developed and produced a novel pumpless, single PDMS-layered microfluidic device (MFD) combined with BMSC-conditioned medium (BMSC-CM) and validated its performance for IVS in this study. We assessed whether the novel system could provide in vitro testicular maturation as measured by increased spermatogenesis and effective testosterone production in vitro. Then, we hypothesized that the newly generated monolayer pumpless MFD based on syngeneic BMSC-CM could support maintenance and expansion of the neonatal mice SSPC pool, spermatogenesis and functional testicular maturation for 42-day-long culture in vitro. We, therefore, generated and validated the syngeneic BMSC-CM pumpless monolayer microfluidic platform for culture of prepubertal C57BL/6 mouse testes by evaluating the increase in epithelial thickness of the seminiferous tubules and luminal diameter, enlargement of the SSPC and differentiating germ cell (GC) pool and measured testosterone levels on days 7, 28 and 42 using flow cytometric (FCM), histologic and mass spectrometric tools.

## Methods

### Study design

**Fig. 1:**
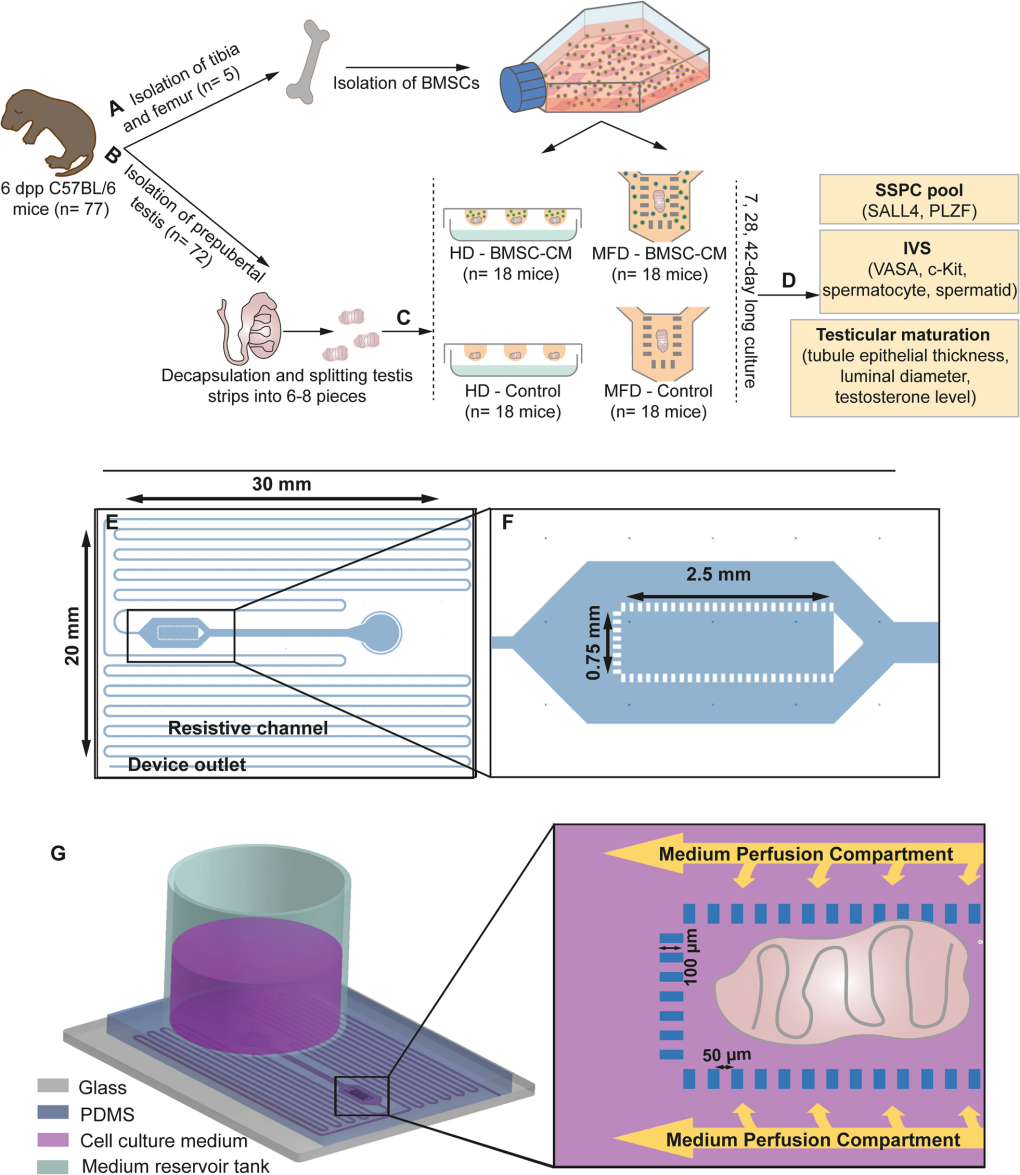
Workflow of the study and design of MFD. **a** Isolation of BMSCs. **b** Testicular tissue collection. **c** Generation of HD and MFD culture systems. **d** Evaluation of SSPC pool, IVS and testicular maturation. **e** Sketch of the mold with resistive channel, device outlet and **f** organ chamber. **g** 3D illustration of MFD with organ placement

The ex vivo experimental study was carried out in compliance with the ARRIVE guideline 2.0. Dependent variables were SSPC pool, IVS and functional testicular maturation parameters; independent variables were time (days 7, 28 and 42) and groups (control and BMSC-conditioned medium, HD and MFD). Experiments were carried out as two parallel and three repeats (Fig. 1a–d).

### Animals

Six days postpartum (dpp) C57BL/6 male mice were purchased from the Experimental Animal Breeding and Application Center (Başkent University, Ankara, Turkey) and served as BMSC and testis donor. The mice were housed in air-conditioned, pathogen-free rooms with a 12/12-h lightning cycle at 20 ± 2 °C and a humidity of 55 ± 5% and fed with a standard mouse diet and tap water ad libitum. The methods used in all animal experiments all conformed to the Guide for the Care and Use of Laboratory Animals and were approved by the Local Ethical Board of Animal Experiments (Hacettepe University, Ankara, Turkey (#52338575-109, September 18, 2018).

### Design, fabrication and validation of MFD

A new pumpless microfluidic platform composed of a medium flow channel (500 µm × 8865 µm, WxL), a core organ chamber, medium perfusion compartments and a highly resistant microchannel region of 150 μm × 500 mm (WxL) was designed. The height of the microchannel network (170 μm) was defined as slightly higher than the achievable thickness of the testicular strips. The organ chamber was fed by two-sided perfusion compartments (each 500 µm in width) through an array of rectangular micropillars. The continuous flow of medium was induced by the hydraulic head at the medium reservoir tank. A long, narrow and necked microchannel region ensured the desired flow rate range by introducing a high resistance to the pumpless flow of the medium (Fig. 1e–g).

Time-dependent flow rate (*Q*) with respect to the hydraulic head of the medium was analytically calculated using MATLAB R2018a. A time-varying dynamic range is obtained roughly between 0.5 and 1 µL/min for passive flow rate control with respect to the change in hydraulic head from 38 to 19 mm (Additional file 1: Info. 1). A 3D computational fluid dynamics (CFD) simulation model was performed to compute the steady-state velocity field distribution inside the microsystem with organ chamber for 1 µL/min by utilizing the laminar flow physic of COMSOL Multiphysics v5.6 software. The surface plot validates that micropillar geometry leads to a lower velocity profile inside the organ chamber respective to the side perfusion channels; thus, microsystem enables low shear stress and a more in vivo*-*like physical environment for the proper growth of the tissue.

The 4-inch silicon master, containing the inverse of the desired geometry, was patterned with deep-reactive ion etching (DRIE), which yielded high aspect ratio microstructuring. For easy peeling off PDMS from the mold, a low energy surface was created by sputtering a chromium layer of 20 nm as an adhesion layer and then a gold layer of 100 nm. Then, the PDMS prepolymer was mixed with the curing agent at a ratio of 10:1, poured on the mold and cured at 80 °C for 2 h following degasification. The PDMS layer was permanently bonded with a glass substrate using O_2_ plasma treatment at 40 W for 60 s. Before bonding, the testicular tissue fragment was placed inside the tissue chamber under a stereomicroscope. The drops of remaining medium containing metabolic by-products were collected from a lateral outlet, defined at the edge of the glass substrate and used for testosterone measurement.

### Isolation and characterization of BMSCs from male mice

BMSCs were isolated from 6-dpp mice (n = 5), as described previously [30, 31]. In brief, after killing by cervical dislocation, tibias and femurs were collected in phosphate-buffered saline (PBS) (Thermo Fisher Scientific, USA) with 1% penicillin–streptomycin (BI, Israel) and flushed with α-minimum essential medium (α-MEM) (BI, USA) with 15% fetal bovine serum (BI, USA) and 1% penicillin–streptomycin, followed by culture at 37 °C, 5% CO_2_. BMSCs were characterized for further experiments by evaluation at passage three of spindle-shaped morphology, differentiation capacity in adipogenic and osteogenic directions and surface antigen analysis by FCM, as described before [30, 32–35]. The BMSCs were distributed as 2 × 10^5^ cell/tube. The cells were immunolabeled with CD44, CD140a, Sca-1 mesenchymal and CD34, CD45 mouse hematopoietic stem cell markers (Additional file 1: Table 1) [36]. Flow cytometric measurements and analyses were performed with a Novocyte (ACEA Biosciences, USA) flow cytometer and Novoexpress 1.3.0. (ACEA Biosciences, USA) software with 10.000 events recorded for each sample.

### Testicular tissue collection and culture

Mice (6-dpp, n = 72) testicles were isolated, decapsulated by removal of the tunica albuginea under a stereomicroscope (Leica, Wetzlar, Germany) with a following division of testes into 8–10 seminiferous tubule fragments (< 1 mm^3^ in size) and placed into organ chamber and wells of MFD and HD, respectively. Testicular strips were grown with 7.5% FBS, 5% KSR and 1% pen-strep supplemented α-MEM medium at 5% CO_2_ at 37 °C. In conditioned media groups, the medium (15% FBS, 1% pen-strep in α-MEM) collected from 24-h culture of BMSCs was mixed with medium including α-MEM with 10% KSR and 1% pen-strep in 1:1 proportion in volume in order to equalize the supplement concentration in culture media of both control and BMSC-CM groups. Culture medium was added into the MFD reservoir every 3 days and was changed every day in the HD setup for 42 days (n = 12, each).

### Flow cytometry

Testicular single cell suspensions from each sample were obtained by chemical digestion with DNAse I (Serva, Israel), 0.25% EDTA–trypsin solution (1:9, v/v) and mechanical disruption through pipetting through 40-μm cell strainers (Corning, USA) [30]. The cells were fixed by 3% PFA and permeabilized by 0.2% Tween 20 in PBS. Then, they were labeled with rabbit-anti-mouse VASA (total GC marker) and PLZF (SSPC marker) and c-Kit (differentiating spermatogonia) antibodies for 30 min at RT. For labeling of VASA, the cells were incubated with a secondary FITC-conjugated goat anti-rabbit IgG antibody (Additional file 1: Table 1) at RT for 30 min. Measurements were taken by Novocyte FCM, and data were analyzed with Novoexpress 1.3.0. software, with 10.000 events recorded for each sample. Immune-positive cells were detected by gating according to unstained and isotype control samples.

### Histomorphometry

Testis fragments were fixed in Bouin’s fixative, underwent tissue processing by dehydration and clearing and were embedded in paraffin. Paraffin sections of 3 µm thickness were obtained, stained with periodic acid Schiff (PAS, Sigma, USA) and evaluated under a light microscope with an attached digital camera (Leica DMR 6000, Germany). Epithelial thickness of the tubules and the luminal diameter of fifty seminiferous tubule cross sections per group were measured using an image analysis program (LASv3 Leica, Germany) [30].

### Immunohistochemistry

An indirect immune peroxidase method was performed on deparaffinized and rehydrated 3-µm-thick sections. Hydrated slides were exposed to H_2_O_2_ for quenching of endogenous peroxidase activity and subjected to heat-induced antigen retrieval with citrate buffer (pH = 6.5). A specific protein blocking was carried out with goat serum (Abcam, USA). The slides were incubated with sequentially an anti-SALL4 primary antibody (1:200) and HRP-goat anti-rabbit IgG secondary antibodies (Additional file 1: Table 1). DAB was applied to samples as chromogen. The slides on which the primary antibody incubation was omitted were used as negative control (Additional file 1: Fig. 1). Hematoxylin was used for counter-staining. The immunolabeled cells were counted in 50 cross sections of seminiferous tubules from each group under a bright-field microscope (Leica DM 6000 BM, Germany) with an analysis program (LASv3 Leica, Germany), as described previously [30].

### Testosterone measurement by LC/MS–MS

The total testosterone concentration in the testicular culture supernatant of HD and MFD setups was assessed using liquid chromatography/mass spectrometry (LC/MS–MS). The 24-h medium samples were collected into separate tubes on days 7, 28 and 42. The 5-µm-sized LC column (C18, XBridge, WATERS, USA) and testosterone standard (T1500, Sigma-Aldrich, USA) were used for operation. The LC/MS–MS analyses were performed with a Xevo TQ-S spectrometer (WATERS, USA).

### Statistical analysis

The normality of the distribution was determined by Shapiro–Wilk. Paired t-test and Wilcoxon signed-rank tests were used for comparison of parametric and nonparametric data, respectively, with a 95% confidence interval. Parametric data were presented as mean ± S.D. Nonparametric data were presented as median, minimum and maximum values. Analyses were performed with the SPSS 23.0 Bivariate Correlation A program.

## Results

### Generation and validation of a novel, single PDMS-layered pumpless MFD

**Fig. 2:**
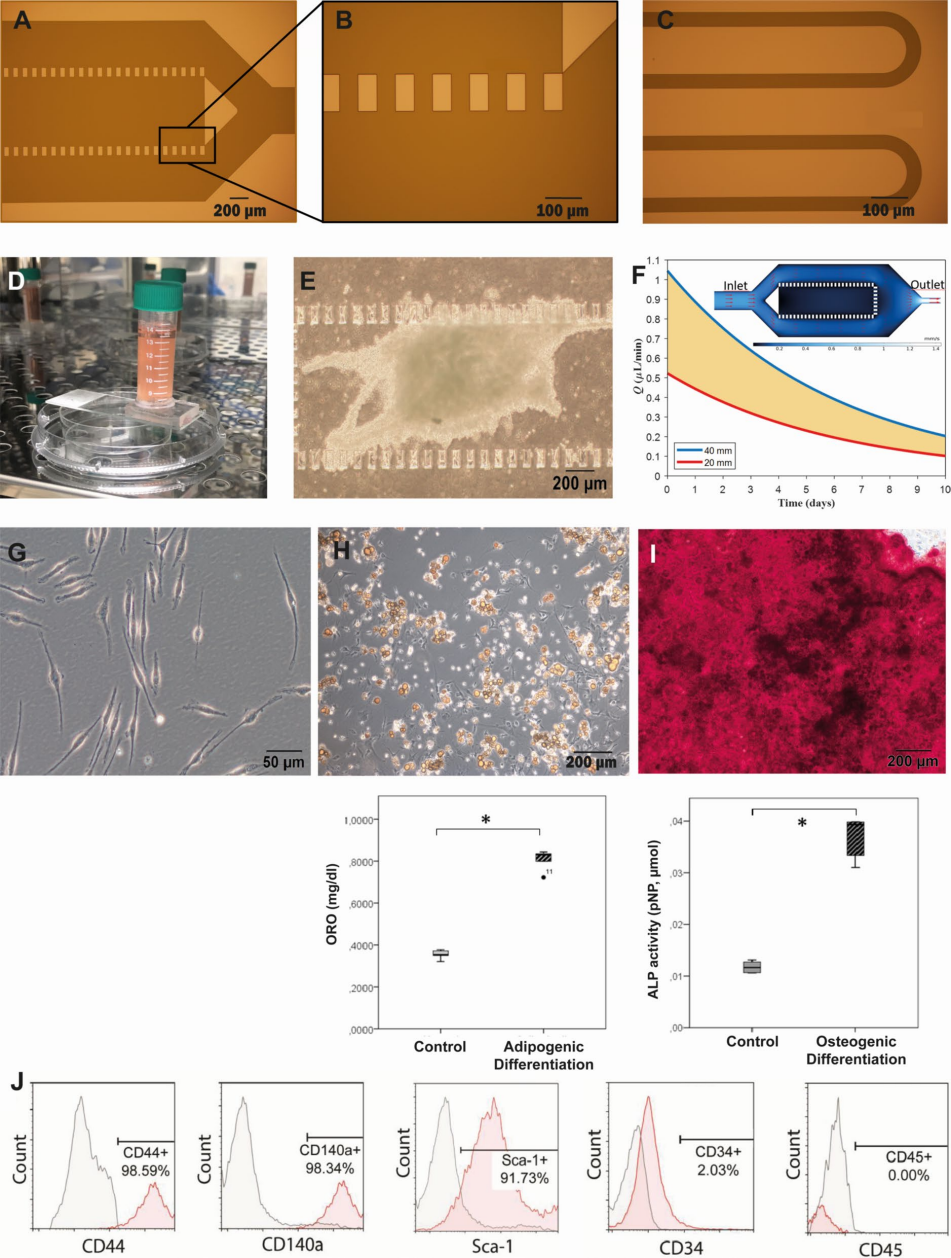
Validation of new MFD and characterization of BMSCs. Micrographs of **a** organ chamber, **b** pillars and **c** resistive channels after photolithography and **d** picture of MFD with BMSC-CM insert. **e** Micrograph of seminiferous tubules within organ chamber during culture. **f** The graph presents the flow rate in resistive channels with different medium levels in the reservoir tank (MATLAB R2018a). Surface plot represents the velocity field inside the microsystem where the red arrows indicate the flow direction (COMSOL Multiphysics v5.6). **g** Micrograph of passage three spindle-shaped BMSCs adhering to culture plate. **h** Adipogenic and **i** osteogenic differentiation of BMSCs (ORO, 20x; ARS, 100x, respectively, *p < 0.01). **j** Characteristic surface antigenic expression of BMSCs by FCM

A profilometer and a digital microscope were used to achieve dimensional accuracy during the microfabrication of the mold (Fig. 2a–c). After generation of a BMSC-CM inlet-inserted MFD (Fig. 2d) and placement of testicular strips (Fig. 2e), a continuous flow was developed within the microchannel by a hydraulic medium head of 38 mm. The flow rate estimated as 0.9 µL/min on average for the first 100 min of the operation by the accumulated volume measurement at the outlet, which is slightly below the numerically achieved flow rate of 0.98 µL/min (Fig. 2f) mainly due to the wafer-level nonuniformity in etch depth. The testicular tissue was immobilized within the chamber for 42 days of culture (patent applications for MFD: #TPTO/2021/004441, #PCT/TR2022/050188). The findings regarding the microfluidic device were presented at the ASRM 2022 Scientific Congress following the national and international patent applications and published in the Fertility & Sterility as Abstract Supplement in Volume 118, Issue 4.

### Characterization of BMSCs

Passage three BMSCs attached to culture plastics and demonstrated a typical spindle–polygonal-shaped morphology (Fig. 2g). Isolated cells underwent adipogenic (Fig. 2h) and osteogenic differentiation (Fig. 2i) successfully. BMSCs homogenously (> 90%) expressed CD44, CD140a and Sca-1 and were negative for hematopoietic markers CD34 and CD45 (< 3%) (Fig. 2j).

### BMSC-CM-enhanced MFD provides maintenance and expansion of the SSPC pool

**Fig. 3:**
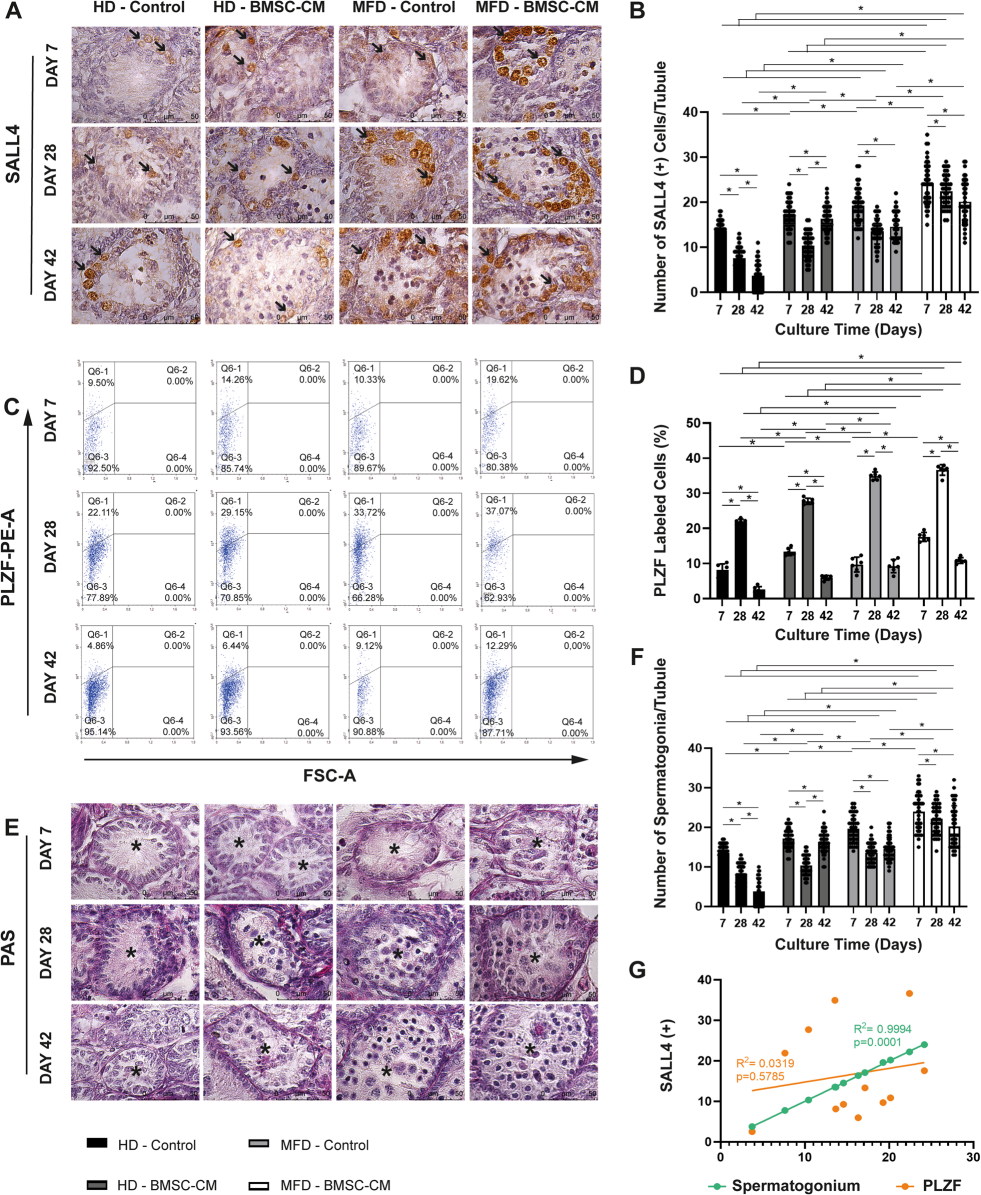
BMSC-contributed MFD provides maintenance and enlargement of SALL4(+) and PLZF(+) SSPC pool and PAS-stained spermatogonia in vitro. Immune labeling of **a** SALL4(+) and **c** PLZF(+) SSPCs, and **e** PAS-stained of spermatogonia in prepubertal mice testes from day 7 to 42 in BMSC-CM-applied and non-applied HD and MFD (SALL4 IHC and PAS–hematoxylin, 1000x). Note the presence of SSPCs indicated by arrow on SALL4-labeled sections. In time change in **b** number of SALL4(+) cells, **d** ratio of PLZF(+) cells to total testicular cells and **f** spermatogonium count in BMSC-CM-applied and non-applied groups are illustrated in bar graph with standard deviation and data distribution (*p < 0.05, n = 6 testes, 50 tubules for SALL4; n = 6 testes for PLZF). **g** Line graph illustrates positive correlation of SALL4 with PLZF labeling and spermatogonium number for SSPCs in control and BMSC groups (*R*^2^ = 0.0319, *p* = 0.5785; *R*^2^ = 0.9994, *p* = 0.001, respectively) (arrows: SSPCs, asterisks: seminiferous tubules)

Addition of BMSC-CM increased the number of SALL4(+) SSPCs (Fig. 3a, b), the PLZF(+) SSPC ratio (Fig. 3c, d) and spermatogonia per tubule (Fig. 3e, f) in the HD setup on days between 7 and 42 for each time point, when compared to non-BMSC controls (*p* = 0.001 for all). Similarly, supplementation of CM increased the number of SSPCs and spermatogonia from day 7 to 42 for each time point, when compared to non-BMSC-CM control group. In terms of the PLZF-labeled SSPC ratio, the BMSC-CM-enhanced MFD was superior to the non-BMSC-CM group on day 7. However, they were similar from this time point forward. The percentage of PLZF-labeled cells in the total testicular cell suspension increased in time from day 7 to 42 in all groups, with an obvious increase in BMSC-CM and/or MFD groups. The BMSC-CM-enhanced MFD was superior to all other groups throughout the experiment, in terms of SSPC pool maintenance and expansion. The number of SALL4(+) SSPCs and PAS-stained spermatogonia decreased between days 7 and 28 (*p* = 0.001) and remained unchanged from this time point forward in BMSC-CM-enhanced and control MFD groups. SALL4(+) SSPC spermatogonial pool presented a constant reduction, when BMSC-CM was not applied in the HD setup from day 7 to 42 (*p* = 0.001). Addition of BMSC-CM not only prevented reduction, but also increased the SALL4(+) SSPC/spermatogonial pool between days 28 and 42 in static HD setup (*p* = 0.001). The number of SALL4(+) cells per tubule presented a strong positive correlation with spermatogonia count and a mild correlation with PLZF-labeled cell ratio (Fig. 3g, *R*^2^ = 0.9994, *p* = 0.001; *R*^2^ = 0.0319, *p* = 0.5785, respectively).

### BMSC-CM-enhanced MFD promotes IVS

**Fig. 4:**
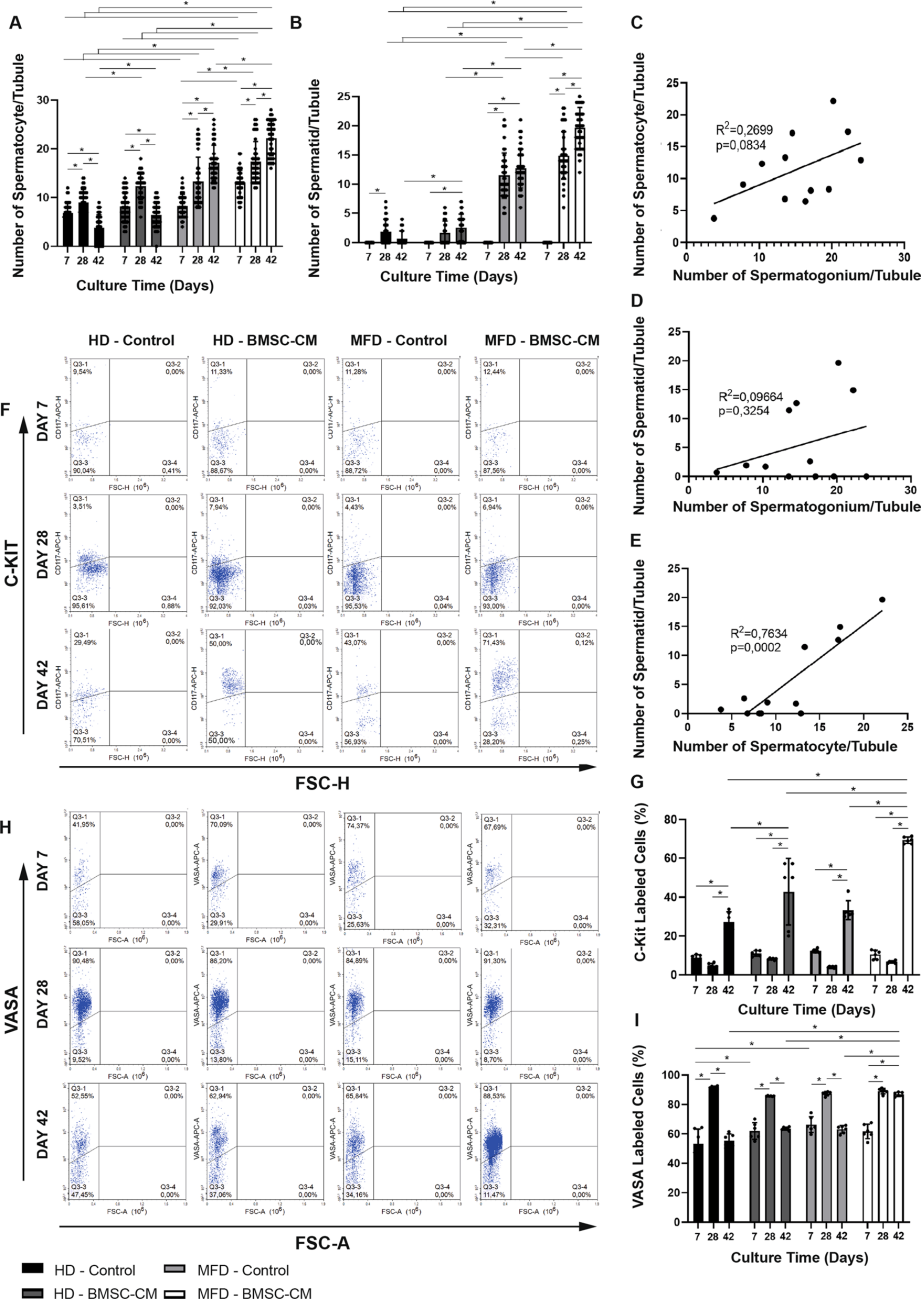
BMSC-CM-contributed MFD promotes IVS. In time change in number of **a** spermatocytes and **b** spermatids per tubules in BMSC-CM-applied and non-applied groups are shown in bar graph with standard deviation and data distribution (*p < 0.05, n = 6 testes, 50 tubules). Line graphs illustrate positive correlation between the **c** number of spermatocyte and spermatogonium, **d** spermatid and spermatogonium and **e** spermatocyte and spermatid in BMSC-CM-applied and non-applied HD and MFD (*R*^2^ = 0.02699, *p* = 0.0894; *R*^2^ = 0.09664, *p* = 0.3254; *R*^2^ = 0.7634, *p* = 0.0002, respectively). Flow cytometric analysis of **f** c-Kit(+) and **h** VASA(+) cell ratio to total testicular cells. Bar graph with standard deviation and data distribution illustrates in time change in **g** c-Kit and **i** VASA-labeled cell ratio to total testicular cells in BMSC-CM-applied and non-applied HD and MFD (*p < 0.05, n = 6 testes)

BMSC-CM increased the number of PAS-stained spermatocytes (Fig. 4a) per seminiferous tubule at all time points in MFD (*p* = 0.001). BMSC-CM also increased the number of round spermatids per tubule on day 42 in HD and on days 28 and 42 in MFD compared to their non-BMSC-CM controls (Fig. 4b). BMSC-enhanced HD was superior to the control group in terms of number of spermatocytes for a 42-day-long period at every time point (*p* = 0.001). The number of spermatids was higher in the BMSC-CM-enhanced HD setup on days 28 and 42, when compared to the non-BMSC-CM control, and they were similar to each other on day 7. The number of spermatocytes was higher in the both the BMSC-CM-enhanced and control MFDs, compared to HD from day 7 to 42 at every time point (*p* = 0.001 for all). The number of spermatids was elevated in both BMSC-CM-enhanced and control MFDs, compared to HD on days 28 and 42 (*p* = 0.001). The BMSC-CM-enhanced MFD was superior to all other groups throughout the experiment in terms of spermatocyte and spermatid numbers except for day 7 for the number of round spermatids, since the spermatid pool was not generated at that time point (*p* = 0.001). The number of spermatocytes increased continuously in BMSC-CM-enhanced and control MFD in progress during the 42-day-long culture period (*p* = 0.001). However, it started to decrease after day 28 in both HD setups with and without BMSC-CM (*p* = 0.001). In the BMSC-CM-enhanced MFD, the number of spermatids increased drastically between days 7 and 42 in a time-dependent manner (*p* = 0.001). The spermatid pool increased between days 7 and 28, but stayed constant after day 28 in control MFD and HD setups. The number of PAS-stained spermatogonium per tubule presented a mild positive correlation with spermatocyte count (Fig. 4c, *R*^2^ = 0.2699, *p* = 0.0834) and weak correlation with number of spermatid (Fig. 4d, *R*^2^ = 0.09664, *p* = 0.3254). There was an overall increase in spermatids from day 7 to 42 with BMSC-CM supplement in the HD platform. The number of spermatid and spermatocyte per tubule demonstrated a strong correlation (Fig. 4e, *R*^2^ = 0.7634, *p* = 0.0002). BMSC-CM supplement furthermore increased the ratio of c-Kit(+) differentiating spermatogonia to total testicular cells and was higher in both MFD and HD setups on day 42 when compared to their respective controls (Fig. 4f, g). The ratio of c-Kit(+) cells in testicles was constant between days 7 and 28, after which it started to increase from day 28 to 42 in both BMSC-CM-enhanced and control MFD and HD setups. The BMSC-CM-enhanced MFD was superior to the other three groups on day 42 (Fig. 4f, g, *p* = 0.001 for all). BMSC-CM also provided a higher VASA(+) total GC number per tubule in MFD and HD setups, when compared to their controls on days 42 and 7, respectively (Fig. 4h, i, p = 0.001). Microfluidic and HD systems with and without BMSC-CM supplementation resulted in similar VASA(+) GC counts on day 28. From day 28 to 42, BMSC-CM supplementation maintained the total GC ratio when applied to MFD, while other groups showed a decline. On day 42, the GC ratio within the testicular cell suspension increased up to 90% in the BMSC-CM-enhanced MFD (*p* = 0.001).

### BMSC-CM-enhanced MFD supports structural and functional maturation of testes

**Fig. 5:**
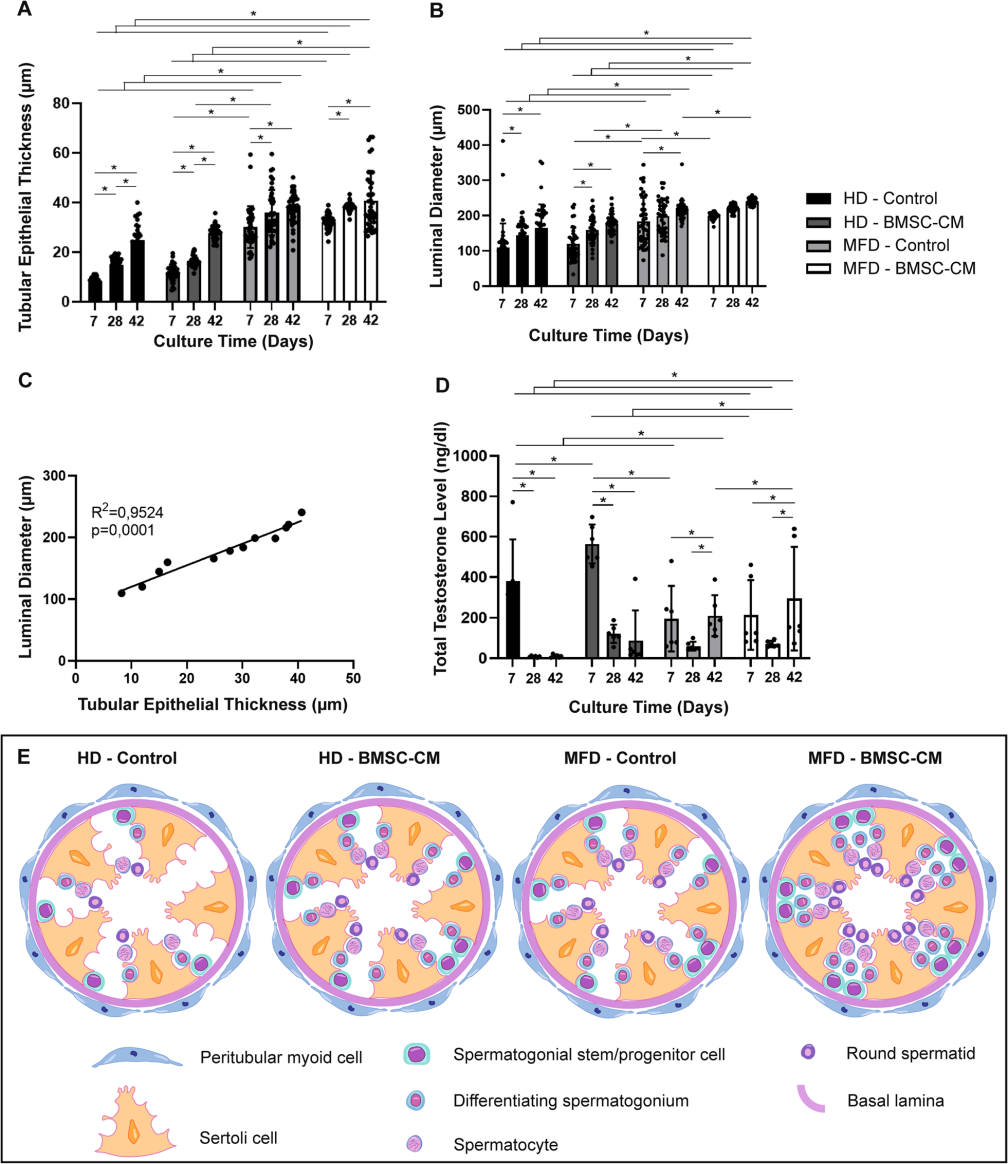
BMSC-CM-contributed MFD supports testicular maturation by increasing tubule epithelial thickness, luminal diameter and testosterone level in vitro*.* Bar graphs illustrate time-dependent change by standard deviation and data distribution of **a** tubule epithelial thickness, **b** luminal diameter and **c** testosterone concentration (*p < 0.05, n = 6 testes, 50 tubules). **d** Line graph illustrates a positive correlation of tubule epithelial thickness with luminal diameter in control and BMSC groups (*R*^2^ = 0.9524, *p* = 0.0001). **e** Illustration of germ cell populations in tubules on day 42

Epithelial thickness of seminiferous tubules and luminal diameter were higher in BMSC-CM-enhanced and control MFDs from day 7 to 42 at every time point when compared to HD setups (Fig. 5a, b, p = 0.001). Epithelial thickness increased in time with and without BMSC-CM HDs. The thickness increased until day 28 and then remained constant from day 28 to 42 in MFDs with and/or without BMSC-CM supplementation. The luminal diameter increased from day 7 to 28 in BMSC-CM-supplemented and control HDs and remained unchanged from this time point forward. The diameter of the lumen was the highest in BMSC-CM MFDs compared to all other groups from day 7 to 42 (*p* = 0.001) (*p* = 0.001). Epithelial thickness of seminiferous tubules presented a strong positive correlation with the luminal diameter (Fig. 5c, *R*^2^ = 0.9524, *p* = 0.0001). BMSC-CM contribution increased testosterone levels in both HD and MFD when compared to their controls on days 7 to 42 (Fig. 5d, p = 0.001 for day 7 in HD, day 42 in MFD; ns for other groups). The total testosterone concentration was higher in both BMSC-CM-supplemented and control HDs compared to MFDs; however, BMSC-CM-supplemented and control MFDs were superior to HDs on day 42 (*p* = 0.001). The total testosterone level presented a sharp decline in both BMSC-CM and other HDs from day 7 to 42 (Fig. 5d, p = 0.001 from day 7 to 28; ns from 28 to 42). Total testosterone concentration decreased also but more mildly from day 7 to 28 (*p* = 0.001) and then raised from day 28 to 42 in both BMSC-applied and non-applied MFDs (*p* = 0.001). Spermatogenic functionality of HD and MFD setups in BMSC-CM-applied and non-applied groups on culture day 42 is summarized by illustration (Fig. 5e).

## Discussion

We designed and produced a single PDMS-layered, pumpless MFD as a novel testicular organ culture platform with a special insert for cell therapy in this study. We successfully validated the new system’s performance on expansion of SSPCs, initiation and maintenance of IVS and progress of functional testicular maturation in a C57BL/6 neonatal mouse model, in which allogeneic BMSCs were used as cellular therapeutics for 42 days. Our microfluidic testis-on-chip system allows a high-quality vision under a microscope with biocompatible single PDMS layer, provides a pumpless medium flow rate between 0.5 and 1.0 µl/min via hydrostatic pressure arising from resistive channels and possesses a cell-based therapy insert for enhancement of spermatogenesis via additional metabolic factors. The flow rate was stabilized nearly to 0.9 µl/min by completing the medium height in reservoir to 38 mm daily. A limited number of studies recently developed microfluidic mouse testis culture platforms, and those devices present slightly different designs with various performances on IVS [17–19]. Komeya et al. generated single [18] and double [19] PDMS-layered MFD with 0.05 µl/min of flow rate by an external syringe pump that had a negative impact in terms of inadequate nutrient and oxygen supply in neonatal mouse testis. The same group modified the device to a two PDMS-layered pumpless format allowing a flow rate between 0.05 and 2.0 µl/min, but the new system exhibited a lower visibility performance under microscope [17]. A similar double-layered MFD by a different group provided a pumpless medium flow (with no numerical data) for adult mouse testis [37]. In our study, we achieved an optimal range for flow rate between 0.5 and 1.0 µl/min in line with previous study [17] without pump. We were able to generate a new, pumpless MFD that offers optimal access to oxygen flow and metabolic supply with a single PDMS layer. Our organ chamber measured 750 µmx2.5 mmx150 µm (WxLxD), and it was bilaterally surrounded by perfusion compartments each 500 µm in width. Tiny, rectangular, fence-like, parallel pillars at the base are designed to ensure gentle fixation of seminiferous tubules within the chamber. The size and configuration of our organ chamber are in line with Yamanaka et al.’s system with a slight decrease in width to increase diffusion efficiency [18]. We achieved the same optimal flow range as Yamanaka’s group by using a single medium reservoir unlike their setup in order to overcome the backflow challenge due to double reservoir. We finally designed a special tube insertion that offers a precision-based assessment of personalized therapeutics, i.e., allogeneic stem cells. Thus, we generated a BMSC-contributed MFD and validated the performance of the new platform for IVS and ex vivo maturation of neonatal mice testis strips for the first time.

Our group previously demonstrated the inductive capacity of allogeneic BMSCs on enlargement of SSC pool and initiation and maintenance of IVS up to round spermatid stage when added to ALI co-culture system [30]; however, the efficiency of static platform was limited due to lack of microvascular flow and inadequate diffusion of nutrients into the organ. Therefore, in this study, we generated a novel pumpless, single PDMS-layered MFD with a special input for cell therapeutics and compared to HD. Here in, we demonstrate that the BMSC-CM-supplemented MFD increased integrity and proliferation of progenitor GC pool in terms of increased number of SALL4(+) SSPCs per tubules and increased ratio of PLZF(+) SSPCs in neonatal mouse testis for 42 days. Both PLZF and SALL4 are highly specific to A_single_, A_paired_ and A_aligned_ spermatogonia as essential transcription factors responsible for maintenance of SSPC pool in mice [38]. The novel MFD increased SALL4(+) SSPC number per tubule by 3.3 and 9 times compared to our previous ALI setup [30] with and without BMSC-CM, respectively. MFD also induced PLZF(+) SSPCs ratio to total testicular cells by 1.5 and 1.7 times when compared to ALI that is set with and without BMSCs in the previous study [30]. The contribution of BMSC to HD provided 1.5 and 3 times increase in PLZF(+) cell percentage on days 28 and 42 and 1.5 times increase in SALL4(+) cell number from day 7 to 42 compared to HD-only setup during ex vivo culture of prepubertal mouse testicular strips. With the contribution of BMSC, HD is able to catch/attain the performance of MFD-alone on day 7 in terms of PLZF and on day 42 in terms of SALL4. The inadequacy of static conditions arising from insufficient nutrition and direct exposure to medium might be covered by addition of mesenchymal paracrine factors. BMSC-contributed HD setup might be considered as a preferable static culture platform since its efficiency is parallel to MFD only. Nevertheless, MFD performed best among ALI and HD and SSPC pool reached its maximum volume in terms of SALL4(+) and PLZF(+) spermatogonia when BMSCs contribute. Our findings related to BMSC mediated in vitro induction of SSPC pool in prepubertal testes are original and establish the precise supporting performance of allogeneic multipotent somatic stromal cells on the complex 3D gonadal niche. This gives strong evidence for the BMSCs to play a major role as an accessory element of testicular stroma and is in line with previous in vivo studies suggesting spermatogonial restoration by intratesticular allogeneic BMSC injection in adult sterile rat and hamster models in vivo [39, 40].

Our monolayered pumpless MFD gave a rate of PLZF immunolabeled cells as nearly 20%, 40% and 12% on days 7, 28 and 42, and it provided 1.5 times increase compared to HD from day 7 to 42 on neonatal testis strips. Our MFD setup also exhibited the number of SALL4(+) SSPCs per tubule as 25.3 ± 2.4, 22.3 ± 2.2 and 20.72 ± 1.2 on days 7 to 42, with 1.5 times enlargement as against to HD from day 7 to 42 in each time point. PMMA/PDMS double-layered pumpless MFD revealed the presence of spermatogonia (with 40% PLZF immune labeling) in tubules after 28 days of follow-up when used with allogeneic neonatal mouse SSC-seeded decellularized testicular tissues. Our 28-day PLZF findings by FCM are in accordance with the previous study. Another double PDMS-layered pumpless MFD provided GFRα1(+) undifferentiated spermatogonia number per tubule as near to 2, and GFRα1(+)/EdU(+) proliferative SSC number per tubule as 1.5 and 1.2 in MFD and ALI platforms, respectively, on day 56 [19]. They evaluated SSCs on day 56 from an interval between 0 and 5 days postnatally; thus, the time period is not precise to be compared with our study. GFRα1 marker labels only the SSCs, including A_single_, A_paired_ and barely A_al4_ spermatogonia, but not A_al8_ and A_al16_ spermatogonia [41]. Though, SALL4 is specific for both the stem and progenitor cell population from A_single_ to A_al16_ in rodents [42]. The number of SALL4(+) SSPCs is naturally higher in our study when compared to GFRα1(+) SSCs and our 42-day time point is earlier than day 56. Though the time point is not the same, the stem cell number is obviously high in our system showing superior performance that could attribute to short width of the organ chamber that may provide higher diffusion capacity comparing to previous system [19]. Taken together, all three studies confirm the good performance of the pumpless systems on the maintenance of spermatogonial pool in vitro. Our study further demonstrates in time change of the whole SSPC pool from day 7 to 42 in a complete mouse spermatogenic cycle that detail the pool enlargement success for possible personalized clinical use such as autologous or allogeneic cell therapies. This study reveals for the first time the efficiency of BMS-CM on the new MFD in terms of induction of SSPC pool in prepubertal testes.

Our recent study revealed a limited capacity of HD culture platform for a complete spermatogenic cycle up to 42 days with enlargement of SSPC pool for 28 days in neonatal mouse testes. Thus, HD allowed a progressive enrichment of SSPC pool from day 7 to 28 with a lower performance than MFD. HD setups maintained the spermatogonial and Sertoli cells in vitrified–warmed adult goat testes [12]; increased OCT4(+), Ki67(+) and AP2-gamma(+) proliferative spermatogonial progenitor cells in gestational 7–12 week human fetal testis [43]; sustained Ki67 and BrdU(+) proliferative GCs with no increase in cCaspase3(+) apoptotic cells from adult human healthy and cancer testicular samples [14] up to 14 days. Our 7- and 28-day results on SSPC amount are parallel to those studies in terms of increase in mitotic GCs shown by different labels. Here in, our HD setup ameliorates the PLZF(+) cell percentage and SALL4(+) cell number on neonatal mouse testis 2 times compared to ALI platform of the previous study from day 7 to 42 [30]. Human [13] and rat [11] neonatal testis ALI setups preserved of PLZF immunolabeled SSPCs up to 50 and 42 days, respectively. Those qualitative results are similar with our 42-day results on HD platform. Our HD setup not only caught the success of ALI, but also demonstrated a better performance in terms of SSPC survival for 42 days that may be due to daily refreshment of medium at high volume (up to 200 µl) when compared to weekly changes at a volume of 1 ml [11, 13, 30]. Thus, we suggest a slightly superior performance of HD platform when compared to ALI for enlargement of SSPC pool in neonatal mice since testicular strips are directly exposed to medium, while diffusion-based nutrition intake is the basic principle in ALI setup. The testis fragments take dome-like shape on agarose gel during the culture in interphase method that causes inadequate diffusion into central regions. HD platform allowed a homogenous distribution of SSPCs in both central and peripheral parts of the strips with no disturbance in testicular shape. This presents strong evidence for HD to be superior to ALI as static culture platform in terms of ex vivo stem/progenitor cell survival in testis. However, HD may not support ex vivo personalized cell therapies properly since the allogeneic BMSC-CM did not even perform well in terms of SSPC pool enlargement compared to MFD according to our data.

Here, we report that BMSC-CM-supported MFD induces both spermatogenesis and testicular maturation in terms of increased percentages of VASA(+) total GCs [44, 45] and c-Kit(+) differentiating spermatogonia, the numbers of spermatocytes and spermatids, and tubular and luminal diameter and testosterone concentration for 42 days. We assessed the proliferation of progenitor cells and onset of meiosis by spermatocyte formation on day 7 (13-dpp) and production of spermatids on day 28 (34-dpp) for a complete spermatogenic cycle in prepubertal C57BL/6 mice [46]. We evaluated maintenance of SSPCs, IVS and testicular maturation until day 42 (48-dpp) in order to interpret post-cycle progression of IVS and to establish whether there is any possible delay in spermatogenic progress toward in vivo development of neonatal mice. Spermatogenesis begins from 7-dpp with the formation of SSPCs and spermatocytes from day 10 to 20 and terminates with formation of spermatids from day 18.8 to 25.3 and sperms on day 34.5 in mice in vivo [47]. The incline of spermatocytes, round spermatids and the total GC number was simultaneous with progressive enlargement of tubules and also with a decline of SSPC pool from day 28 and 42 that show the spermatogonial stem cells entering meiotic cycle. Significant augmentation in testosterone levels confirmed the functional testicular maturation within the same period. Allogeneic BMSCs presented an inferior performance on the HD platform when compared to MFD in terms of spermatogenic differentiation with decreasing number of VASA(+) GCs and testicular maturation with a lesser tubular enlargement and a lower testosterone level measurements from day 28 to 42. Allogeneic BMSCs were not able to increase neonatal mice VASA(+) cells from day 28 to 42 when applied to ALI in our previous study [30] revealing their limited induction capacity on both ALI and HD static setups. Recent BMSC-contributed MFD noticeably improved spermatogenesis and functional maturation of testis by a nearly 2 times increase in number of spermatocytes, spermatids, ratio of c-Kit(+) and VASA(+) cells and tubular enlargement compared to ALI and HD platforms with BMSCs on day 28 of culture. BMSC-CM-supplemented MFD demonstrated a 4 times higher performance compared to ALI and HD on day 42. These results clearly suggest that allogeneic BMSCs perform better in the vicinity of the microvascular circulation, which might provide an optimal and controlled tubular distribution of stem cell secretome compared to simple diffusion of static culture setups. Therefore, the new single PDMS-layered pumpless MFD may present an effective solution for prepubertal male infertility as a tool to precisely apply allogeneic/autologous BMSC and BMSC-CM therapy by mimicking the physiological testicular niche conditions ex vivo.

Our novel pumpless single PDMS-layered MFD is comparable in terms of IVS and testicular functional maturation to Komeya’s 2 PDMS-layered syringe pumped [19] and/or pumpless [17] devices that contributed to a long-term *Acr-Gfp* transgenic neonatal mice testicular culture with successful spermatogenic differentiation in vitro. Our system increased the number of spermatocyte by 25% and spermatid by 30% on 50 seminiferous tubules and offered a ratio of VASA(+) total GCs up to 90% from day 7 to 28 maintained until day 42. A 70% increase in c-Kit(+) differentiating spermatogonia with high testosterone levels (60.5 ~ 80.4 ng/dl on day 28 and 212.3 ~ 298.7 ng/dl on day 42) confirmed progressive induction of IVS and functional maturation of testis in the same period. A two PDMS-layered pumped MFD [19] increased the ratio of GFP-expressing acrosin(+) to total seminiferous tubular area up to 50% from day 21 to 28 and then a constant expression. The study also reported the presence of SCP3(+) spermatocytes and PNA(+) spermatids (timepoint was unspecified) with a testosterone range between 2.4 and 24.0 μg/day/g testis (0.6–5.7 ng/dl) in 2 samples (from 4 measurements) on days 28 and 35 of culture. Our 28- to 42-day induction findings on differentiating GC maintenance and testosterone production contribute to a longer-term capacity of new pumpless MFD when compared to previous pumped system. Our findings are in line with Komeya group’s pumpless device that maintained 90% of Acr-GFP(+) and TRA98(+) tubules within the neonatal transgenic mice testis on week 12 [17]. In our study, the flow rate has been strictly fixed to 0.9 µl/min by daily addition of medium to keep the tank height as 38 mm. Komeya’s pumpless MFD give a flow rate range between 0.05 and 2.0 µl/min and the pumped system a fixed flow rate as 0.05 µl/min. The higher stabilized pumpless flow rate in the recent platform might lead to further improvement in IVS after day 28 and the higher amount of testosterone production in our study compared to previous systems. Therefore, our platform successfully enlarged neonatal mouse SSPC pool and induced spermatogenesis from SSCs up to haploid spermatids for 42 days by efficient production of testosterone. Komeya’s pumped [19], but not the pumpless [17] MFD has been able to produce both round/elongated spermatids and further functional performance of those haploid cells was assessed by microinsemination to adult female ICR mice in which healthy offspring were collected in a longer period comparing to our study. Our group’s results are limited to 42-day-long performance of the novel pumpless MFD until haploid GC formation; however, it comprises a detailed phenotypic classification of enlarging SSPC pool and differentiating spermatogonial cells for a whole spermatogenic cycle. This study also reveals the variation in different stage GC populations throughout a complete spermatogenic cycle and shows the efficiency of the new single PDMS-layered pumpless system. BMSC-CM supplementation maximized the efficiency of the device on induction of testicular maturation including the proliferation and differentiation of SSPCs.

The generated MFD using an insert for BMSC-CM as therapeutic in a pumpless and monolayered platform induced enlargement and maintenance of the SSPC pool, testicular maturation and spermatogenesis up to round spermatids in prepubertal newborn C57BL/6 mice for 42 days in vitro. The secretory potential of BMSCs maximized the efficiency of the microfluidic system in terms of male GC production ex vivo. However, the results are limited to in vitro conditions and need to be validated in vivo in microinseminated adult female mice following further cycles of spermatogenesis. Meanwhile, the obtained GCs should be tested in terms of genetic integrity during fertilization and embryonic development of offspring. However, the new single PDMS-layered pumpless MFD demonstrated a clear optimal performance ex vivo in terms of normal distribution and statistical relevance of the obtained data and confirmed accurate flow rate that is assisted to tubular growth. Our validation results are reliable since we classified subpopulations of GCs multiple specific phenotypic markers [17, 19, 48], simultaneously assessed the testosterone production levels by LC/MS–MS [19] and tubular growth by full-quantitative histomorphometry [35, 49]. Moreover, the study presents the first and preliminary demonstration of BMSC-CM performance on IVS and may be a strong candidate for personalized male infertility treatment in the andrology clinic. On the other hand, allogeneic and/or autologous BMSCs may still have unknown risks that need to be eliminated before translation to the clinics. The mutagenicity and metabolic content of mesenchymal secretome should be analyzed and precisely optimized in further studies prior to introduction to clinic.

## Conclusion

The regenerative potential of BMSC is sustained by a new microfluidic system with a single PDMS layer, which does not require a pump and also provides high-quality imaging. The new platform induced testicular growth, self-renewal of SSPC and differentiation of spermatogenic cells up to round spermatids for a whole cycle ex vivo. We applied BMSC-CM as a model therapeutic in the current study, whereas the generated MFD might be used as a high-technology platform for validation of different personalized cell-based therapeutic agents in order to cure prepubertal and adult infertility cases. Furthermore, the new theragnostic microfluidic platform may contribute to a safe, precision-based cell and tissue banking protocol for prepubertal fertility restoration in future.

## Supplementary Information

**Additional file 1: Supplementary Table 1**. The information of antibodies which were used in IHC and FCM.** Supplementary Information 1**. The calculation of time-dependent flow rate with respect to the height of culture medium based on Hagen–Poiseuille equation.** Supplementary Figure 1**. Negative control micrograph presenting noni-mmunolabelling for the SALL4 SSPC marker (1000x).

## Data Availability

The data from this study are available from the corresponding author upon reasonable request.

## Abbreviations

SSC: Spermatogonial stem cell
SSPC: Spermatogonial stem/progenitor cell
GC: Germ cell
IVS: In vitro Spermatogenesis
PDMS: Polydimethylsiloxane
MFD: Microfluidic device
ALI: Air–liquid interphase
BMSC: Bone marrow-derived mesenchymal stem cell
BMSC-CM: Bone marrow-derived mesenchymal stem cell-conditioned medium
LC/MS–MS: Liquid chromatography/mass spectrometry- mass spectrometry
KSR: Knockout serum replacement
FBS: Fetal bovine serum
PBS: Phosphate buffer saline
Pen-strep: Penicillin–streptomycin
HD: Hanging Drop
FCM: Flow cytometry
CFD: Computational fluid dynamics
*Q*: Flow rate
DRIE: Deep-reactive ion etching
dpp: Days postpartum
PAS: Periodic Acid Schiff

## References

[1] ACS. Key Statistics for Childhood Cancers; 2022. https://www.cancer.org/cancer/cancer-in-children/key-statistics.html.

[2] Pco ASRM (2019). Fertility preservation in patients undergoing gonadotoxic therapy or gonadectomy: a committee opinion. Fertil Steril.

[3] ASRM ECo (2018). Fertility preservation and reproduction in patients facing gonadotoxic therapies: an Ethics Committee opinion. Fertil Steril.

[4] Wasilewski-Masker K, Seidel KD, Leisenring W, Mertens AC, Shnorhavorian M, Ritenour CW (2014). Male infertility in long-term survivors of pediatric cancer: a report from the childhood cancer survivor study. J Cancer Surv.

[5] Siegel RL, Miller KD, Fuchs HE, Jemal A (2022). Cancer statistics, 2022. CA Cancer J Clin.

[6] Yersal N, Köse S, Horzum U, Özkavukcu S, Orwig KE, Korkusuz P (2020). Leptin promotes proliferation of neonatal mouse stem/progenitor spermatogonia. J Assist Reprod Genet.

[7] Medrano JV, Rombaut C, Simon C, Pellicer A, Goossens E (2016). Human spermatogonial stem cells display limited proliferation in vitro under mouse spermatogonial stem cell culture conditions. Fertil Steril.

[8] Gholami K, Vermeulen M, Del Vento F, de Michele F, Giudice MG, Wyns C (2020). The air-liquid interface culture of the mechanically isolated seminiferous tubules embedded in agarose or alginate improves in vitro spermatogenesis at the expense of attenuating their integrity. In Vitro Cell Dev Biol Anim.

[9] Yokonishi T, Sato T, Katagiri K, Ogawa T (2013). In vitro spermatogenesis using an organ culture technique. Methods Mol Biol.

[10] Sato T, Katagiri K, Gohbara A, Inoue K, Ogonuki N, Ogura A (2011). In vitro production of functional sperm in cultured neonatal mouse testes. Nature.

[11] Matsumura T, Sato T, Abe T, Sanjo H, Katagiri K, Kimura H (2021). Rat in vitro spermatogenesis promoted by chemical supplementations and oxygen-tension control. Sci Rep.

[12] Patra T, Pathak D, Gupta MK (2021). Comparison of two culture methods during in vitro spermatogenesis of vitrified-warmed testis tissue: Organ culture vs. hanging drop culture. Cryobiology.

[13] Yuan Y, Li L, Cheng Q, Diao F, Zeng Q, Yang X (2020). In vitro testicular organogenesis from human fetal gonads produces fertilization-competent spermatids. Cell Res.

[14] Jørgensen A, Young J, Nielsen JE, Joensen UN, Toft BG, Rajpert-De Meyts E (2014). Hanging drop cultures of human testis and testis cancer samples: a model used to investigate activin treatment effects in a preserved niche. Br J Cancer.

[15] de Michele F, Poels J, Vermeulen M, Ambroise J, Gruson D, Guiot Y (2018). Haploid germ cells generated in organotypic culture of testicular tissue from prepubertal boys. Front Physiol.

[16] Gholami K, Pourmand G, Koruji M, Ashouri S, Abbasi M (2018). Organ culture of seminiferous tubules using a modified soft agar culture system. Stem Cell Res Ther.

[17] Komeya M, Hayashi K, Nakamura H, Yamanaka H, Sanjo H, Kojima K (2017). Pumpless microfluidic system driven by hydrostatic pressure induces and maintains mouse spermatogenesis in vitro. Sci Rep.

[18] Yamanaka H, Komeya M, Nakamura H, Sanjo H, Sato T, Yao M (2018). A monolayer microfluidic device supporting mouse spermatogenesis with improved visibility. Biochem Biophys Res Commun.

[19] Komeya M, Kimura H, Nakamura H, Yokonishi T, Sato T, Kojima K (2016). Long-term ex vivo maintenance of testis tissues producing fertile sperm in a microfluidic device. Sci Rep.

[20] Zhankina R, Baghban N, Askarov M, Saipiyeva D, Ibragimov A, Kadirova B (2021). Mesenchymal stromal/stem cells and their exosomes for restoration of spermatogenesis in non-obstructive azoospermia: a systemic review. Stem Cell Res Ther.

[21] Sagaradze G, Basalova N, Kirpatovsky V, Ohobotov D, Nimiritsky P, Grigorieva O (2019). A magic kick for regeneration: role of mesenchymal stromal cell secretome in spermatogonial stem cell niche recovery. Stem Cell Res Ther.

[22] Qian C, Meng Q, Lu J, Zhang L, Li H, Huang B (2020). Human amnion mesenchymal stem cells restore spermatogenesis in mice with busulfan-induced testis toxicity by inhibiting apoptosis and oxidative stress. Stem Cell Res Ther.

[23] Lu J, Liu Z, Shu M, Zhang L, Xia W, Tang L (2021). Human placental mesenchymal stem cells ameliorate chemotherapy-induced damage in the testis by reducing apoptosis/oxidative stress and promoting autophagy. Stem Cell Res Ther.

[24] Sherif IO, Sabry D, Abdel-Aziz A, Sarhan OM (2018). The role of mesenchymal stem cells in chemotherapy-induced gonadotoxicity. Stem Cell Res Ther.

[25] Hassan AI, Alam SS (2014). Evaluation of mesenchymal stem cells in treatment of infertility in male rats. Stem Cell Res Ther.

[26] Chen Y-T, Chuang F-C, Yang C-C, Chiang JY, Sung P-H, Chu Y-C (2021). Combined melatonin-adipose derived mesenchymal stem cells therapy effectively protected the testis from testicular torsion-induced ischemia-reperfusion injury. Stem Cell Res Ther.

[27] Zhong L, Yang M, Zou X, Du T, Xu H, Sun J (2020). Human umbilical cord multipotent mesenchymal stromal cells alleviate acute ischemia-reperfusion injury of spermatogenic cells via reducing inflammatory response and oxidative stress. Stem Cell Res Ther.

[28] Hsiao C-H, Ji AT-Q, Chang C-C, Chien M-H, Lee L-M, Ho JH-C (2019). Mesenchymal stem cells restore the sperm motility from testicular torsion-detorsion injury by regulation of glucose metabolism in sperm. Stem Cell Res Ther.

[29] Hsiao C-H, Ji AT-Q, Chang C-C, Cheng C-J, Lee L-M, Ho JH-C (2015). Local injection of mesenchymal stem cells protects testicular torsion-induced germ cell injury. Stem Cell Res Ther.

[30] Önen S, Köse S, Yersal N, Korkusuz P (2022). Mesenchymal stem cells promote spermatogonial stem/progenitor cell pool and spermatogenesis in neonatal mice in vitro. Sci Rep.

[31] Huang S, Xu L, Sun Y, Wu T, Wang K, Li G (2015). An improved protocol for isolation and culture of mesenchymal stem cells from mouse bone marrow. J Orthop Transl.

[32] Sareen N, Sequiera GL, Chaudhary R, Abu-El-Rub E, Chowdhury SR, Sharma V (2018). Early passaging of mesenchymal stem cells does not instigate significant modifications in their immunological behavior. Stem Cell Res Ther.

[33] Drela K, Stanaszek L, Nowakowski A, Kuczynska Z, Lukomska B (2019). Experimental strategies of mesenchymal stem cell propagation: adverse events and potential risk of functional changes. Stem Cells Int.

[34] Yang YK, Ogando CR, Wang See C, Chang TY, Barabino GA (2018). Changes in phenotype and differentiation potential of human mesenchymal stem cells aging in vitro. Stem Cell Res Ther.

[35] Tamadon A, Mehrabani D, Rahmanifar F, Jahromi AR, Panahi M, Zare S (2015). Induction of spermatogenesis by bone marrow-derived mesenchymal stem cells in Busulfan-induced azoospermia in hamster. Int J Stem Cells.

[36] Houlihan DD, Mabuchi Y, Morikawa S, Niibe K, Araki D, Suzuki S (2012). Isolation of mouse mesenchymal stem cells on the basis of expression of Sca-1 and PDGFR-alpha. Nat Protoc.

[37] Naeemi S, Sabetkish S, Kiani MJ, Dehghan A, Kajbafzadeh AM. Ex-vivo and in-vivo expansion of spermatogonial stem cells using cell-seeded microfluidic testis scaffolds and animal model. Cell Tissue Bank. 2022. 10.1007/s10561-022-10024-6 35792989

[38] Hobbs RM, Fagoonee S, Papa A, Webster K, Altruda F, Nishinakamura R (2012). Functional antagonism between Sall4 and Plzf defines germline progenitors. Cell Stem Cell.

[39] Monsefi M, Fereydouni B, Rohani L, Talaei T (2013). Mesenchymal stem cells repair germinal cells of seminiferous tubules of sterile rats. Iran J Reprod Med.

[40] Vahdati A, Fathi A, Hajihoseini M, Aliborzi G, Hosseini E (2017). The regenerative effect of bone marrow-derived stem cells in spermatogenesis of infertile hamster. World J Plast Surg.

[41] Ibtisham F, Honaramooz A (2020). Spermatogonial stem cells for in vitro spermatogenesis and in vivo restoration of fertility. Cells.

[42] Tseng Y-T, Liao H-F, Yu C-Y, Mo C-F, Lin S-P. Epigenetic factors in the regulation of prospermatogonia and spermatogonial stem cells. Reproduction (Cambridge, England). 2015;150. 10.1530/REP-14-0679 26116003

[43] Jørgensen A, Nielsen JE, Perlman S, Lundvall L, Mitchell RT, Juul A (2015). Ex vivo culture of human fetal gonads: manipulation of meiosis signalling by retinoic acid treatment disrupts testis development. Hum Reprod.

[44] Castrillon DH, Quade BJ, Wang TY, Quigley C, Crum CP (2000). The human VASA gene is specifically expressed in the germ cell lineage. Proc Natl Acad Sci USA.

[45] Tanaka SS, Toyooka Y, Akasu R, Katoh-Fukui Y, Nakahara Y, Suzuki R (2000). The mouse homolog of Drosophila Vasa is required for the development of male germ cells. Genes Dev.

[46] Cooke H, Saunders P (2002). Mouse models of male infertility. Nat Rev Genet.

[47] Margolin G, Khil PP, Kim J, Bellani MA, Camerini-Otero RD (2013). Integrated transcriptome analysis of mouse spermatogenesis. BMC Genom.

[48] Robinson M, Bedford E, Witherspoon L, Willerth SM, Flannigan R (2022). Using clinically derived human tissue to 3-dimensionally bioprint personalized testicular tubules for in vitro culturing: first report. F S Sci.

[49] Montoto LG, Arregui L, Sanchez NM, Gomendio M, Roldan ER (2012). Postnatal testicular development in mouse species with different levels of sperm competition. Reproduction.

