# Supplementary material for: A pumpless monolayer microfluidic device based on mesenchymal stem cell-conditioned medium promotes neonatal mouse in vitro spermatogenesis
Source: Stem Cell Res Ther. 2023 May 11;14:127. doi: 10.1186/s13287-023-03356-x (PMC10173473; doi:10.1186/s13287-023-03356-x)
Supplement: Supplementary file 1 — Additional file 1: Supplementary Table 1. The information of antibodies which were used in IHC and FCM. Supplementary Information 1. The calculation of time-dependent flow rate with respect to the height of culture medium based on Hagen–Poiseuille equation. Supplementary Figure 1. Negative control micrograph presenting noni-mmunolabelling for the SALL4 SSPC marker (1000x). [file 13287_2023_3356_MOESM1_ESM.docx]

**SUPPLEMENTAL MATERIAL**

**Supplementary Table 1.** The information of antibodies which were used in IHC and FCM.

| Antibody/Labelling Kit | Conjugate | Isotype Control | Host | Clone | Manufacturer | Catalog # |
| --- | --- | --- | --- | --- | --- | --- |
| Anti-mouse PLZF | PE | PE-Mouse IgG1, κ | Rat | Monoclonal | BD | 564850 |
| Anti-mouse c-Kit | APC | APC-Rat IgG2b, κ | Rat | Monoclonal | BD | 553356 |
| Anti-mouse DDX4-MVH/VASA | Unconjugated | FITC- Rat IgG2b | Rabbit | Monoclonal | Invitrogen | MA5-15565 |
| Anti-mouse CD44 | PE | PE- Rat IgG2b | Rat | Monoclonal | BD | 561860 |
| Anti-mouse CD140a | APC | APC-Rat IgG2a | Rat | Monoclonal | BD | 562777 |
| Anti-mouse Sca-1 | PE | PE-Rat IgG2a | Rat | Monoclonal | BD | 560654 |
| Anti-mouse CD34 | PE | PE-Rat IgG2a | Rat | Monoclonal | BD | 551387 |
| Anti-mouse CD45 | FITC | FITC- Rat IgG2b | Rat | Monoclonal | BD | 561088 |
| Anti-mouse SALL4 | Unconjugated | - | Rabbit | Monoclonal | Abcam | ab29112 |
| Rabbit HRP/DAB IHC Detection Kit | - | - | Goat | Monoclonal | Abcam | ab236466 |

**Supplementary Information 1.**

Time-dependent flow rate (*Q*) with respect to the height of culture medium were analytically calculated based on Hagen–Poiseuille equation (1) for the given dimensions of resistive channel region using MATLAB. Here, hydraulic resistances (*R*_h_) of the tissue chamber, medium perfusion compartments, and medium flow channel are ignored because their resistances are significantly lower respective to that of the resistive microchannel region. Equations 1 and 2 are obtained as follows where *μ* and *ρ* are the dynamic viscosity (Pa⸱s) and density (kg/m^3^) of cell culture medium; *h*_0_ is the initial hydraulic-head length (m) at the medium reservoir tank; *w*, *h* and *L* are the width (m), height (m) and length (m) of the resistive microchannel; *A* and *g* are the area of the tube cross-section (m^2^) and the acceleration of gravity (m/s^2^), respectively.


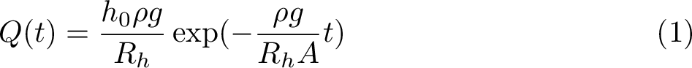


[1]


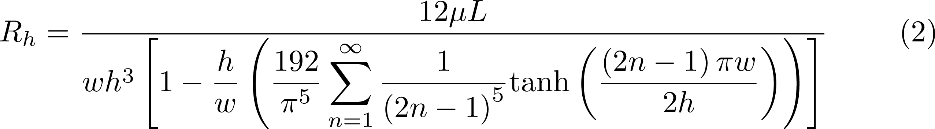


[2]


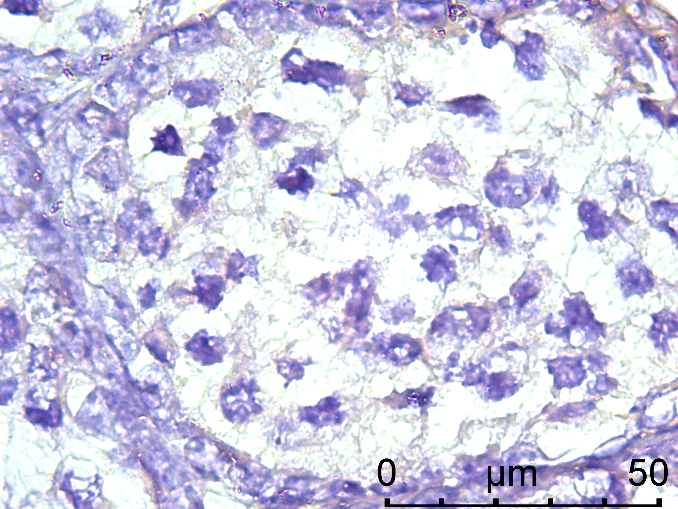


**Supplementary Figure 1.** Negative control micrograph presenting non-immunolabelling for the SALL4 SSPC marker (1000x).
